# Angiogenic mRNA and microRNA Gene Expression Signature Predicts a Novel Subtype of Serous Ovarian Cancer

**DOI:** 10.1371/journal.pone.0030269

**Published:** 2012-02-13

**Authors:** Stefan Bentink, Benjamin Haibe-Kains, Thomas Risch, Jian-Bing Fan, Michelle S. Hirsch, Kristina Holton, Renee Rubio, Craig April, Jing Chen, Eliza Wickham-Garcia, Joyce Liu, Aedin Culhane, Ronny Drapkin, John Quackenbush, Ursula A. Matulonis

**Affiliations:** 1 Department of Biostatistics and Computational Biology, Dana-Farber Cancer Institute, Boston, Massachusetts, United States of America; 2 Department of Cancer Biology, Dana-Farber Cancer Institute, Boston, Massachusetts, United States of America; 3 Illumina, Inc., San Diego, California, United States of America; 4 Department of Pathology, Division of Woman's and Perinatal Pathology, Brigham and Women's Hospital, Boston, Massachusetts, United States of America; 5 Department of Medical Oncology, Dana-Farber Cancer Institute, Boston, Massachusetts, United States of America; 6 Harvard School of Public Health, Boston, Massachusetts, United States of America; 7 Harvard Medical School, Boston, Massachusetts, United States of America; Baylor College of Medicine, United States of America

## Abstract

Ovarian cancer is the fifth leading cause of cancer death for women in the U.S. and the seventh most fatal worldwide. Although ovarian cancer is notable for its initial sensitivity to platinum-based therapies, the vast majority of patients eventually develop recurrent cancer and succumb to increasingly platinum-resistant disease. Modern, targeted cancer drugs intervene in cell signaling, and identifying key disease mechanisms and pathways would greatly advance our treatment abilities. In order to shed light on the molecular diversity of ovarian cancer, we performed comprehensive transcriptional profiling on 129 advanced stage, high grade serous ovarian cancers. We implemented a, re-sampling based version of the ISIS class discovery algorithm (rISIS: robust ISIS) and applied it to the entire set of ovarian cancer transcriptional profiles. rISIS identified a previously undescribed patient stratification, further supported by micro-RNA expression profiles, and gene set enrichment analysis found strong biological support for the stratification by extracellular matrix, cell adhesion, and angiogenesis genes. The corresponding “angiogenesis signature” was validated in ten published independent ovarian cancer gene expression datasets and is significantly associated with overall survival. The subtypes we have defined are of potential translational interest as they may be relevant for identifying patients who may benefit from the addition of anti-angiogenic therapies that are now being tested in clinical trials.

## Introduction

Advanced epithelial ovarian cancer is notable for initial sensitivity to platinum- and taxane-based chemotherapy [1], [2], but the vast majority of women will develop recurrent ovarian cancer within 12 to 24 months and will eventually die from increasingly platinum- and chemotherapy-resistant disease. One possible reason that ovarian cancer remains refractory to therapy is that there are distinct molecular subtypes, which different cellular properties, each of which may require different therapeutic approaches to effectively treat the disease.

Gene expression profiling data represents the largest source of genomic data that might be of use in identifying clinically-relevant subtypes in ovarian cancer, and multiple studies have explored its use for finding predictive biomarkers and clinically-relevant subtypes in ovarian cancer [3], [4], [5], [6], [7], [8], [9], [10], [11]. Tothill et al. [10] used an unsupervised clustering of gene expression profiles and proposed the existence of six subtypes in epithelial ovarian cancer (denoted C1–C6) and a seventh group of unclassifiable tumors (NC); the C1 subtype, which had the poorest prognosis, was found to be characterized by expression of a responsive stromal signature. Dressman and colleagues [5] used a supervised statistical approach to predict response to platinum-based treatment from gene expression data; they found evidence linking chemoresistance to Src and Rb/E2F pathway activity. Recently the “The Cancer Genome Atlas” (TCGA, http://cancergenome.nih.gov) consortium released a set of 500 gene expression profiles from 500 serous ovarian cancer tumor samples that they used to infer the existence of multiple subtypes [12]. However, none of the subtypes identified to date have seen widespread clinical application and often fail to validate in independent datasets.

Our goal was to identify robust molecular subtypes of high-grade serous ovarian cancer and sets of functionally defined classification genes that might give insight into potential therapies. We began with a collection of 129 clinically-annotated formalin-fixed, paraffin-embedded (FFPE) FIGO stage III and stage IV high grade serous ovarian samples previously used to construct a tissue microarray [13], [14] and used the Illumina DASL™ BeadArray™ platform to profile mRNA expression in these patients; in parallel, we profiled the expression level of 743 non-coding micro-RNAs.

Having collected and normalized the gene expression data, we ran the rISIS class discovery algorithm [15] and subjected the resulting candidate subtypes to a rigorous validation and evaluation scheme including bootstrap based stability evaluation and integration of microRNA profiles, and then validated the resulting subtypes and associated gene signature on ten independent gene expression data sets representing data from 1,606 ovarian cancer patients.

## Methods

### Patient identification

Approval was obtained from the Dana-Farber/Harvard Cancer Center Institutional Review Board (IRB) to review all pathology reports between January 1999 and December 2005 in the Brigham and Women's Hospital Department of Pathology database that included the diagnosis of “ovarian cancer” and collect clinical data associated with those patients. Eligible patients had a diagnosis of late stage (all FIGO stage III–IV except 1 case of IIc) high grade papillary serous ovarian carcinoma, pathology blocks available for generation of a high-density tissue microarray (HTMA) [13]. Patient clinical and demographic characteristics were extracted including: age at diagnosis, stage of disease, surgical procedures, chemotherapy treatment given, response to chemotherapy, date of diagnosis, date of first disease recurrence, and date of death or last documented visit to a medical provider.

### RNA extraction and microarray hybridization

RNA was extracted from FFPE blocks originally used for TMA construction. H&E slides were reviewed by a dedicated gynecologic pathologist (MSH), and three 0.8 mm tissue cores were taken from the corresponding FFPE samples at locations adjacent to the original TMA cores; selected areas for sampling were based on having low levels of infiltrating, necrotic, or other contaminating non-tumor tissue. Messenger RNA was extracted using the Qiagen RNeasy FFPE kit with; RNA quality and integrity was assessed using profiles on the Agilent BioAnalyzer and 129 samples passing this basic QC analysis were analyzed using a prototype Illumina DASL BeadArray containing approximately 12,000 selected mRNAs (ArrayExpress Array Design Accession A-MEXP-931) [16]; twelve samples were run in duplicate to allow estimation of the reproducibility of the assay. In addition, we used a prototype DASL-based microRNA expression profiling BeadArray containing probes to 743 microRNAs (ArrayExpress Array Design Accession A-MEXP-1678) [17] to survey patterns of microRNA activity in the samples. The resulting data were normalized using a variance stabilizing transformation combined with quantile normalization as implemented in the Bioconductor lumi package [18], [19]. Both data sets were submitted to the ArrayExpress data repository (ArrayExpress Experiment Accession E-MTAB-386).

### Data analysis

To identify ovarian cancer subtypes, the 129 tumor samples were divided into a training set (n = 82) and a model selection set (n = 47). Genes on the microarray were filtered to select the 1000 most highly variable in their expression levels across samples but which also had low variability among the twelve sets of duplicates. We used the rISIS class discovery algorithm with these 1000 genes to identify distinct partitions of the 82 training samples and to select the 100 genes that provided the most significant statistical support for the partition. We then tested our initial candidate set of partitions against the 47 model selection test set of samples and retained four that retained statistical significance (classification stability >95% during bootstrap) in the model selection set.

MicroRNA expression data from 743 known human miRNAs profiled in the same 129 tumor samples were tested using PAM (predictive analysis of microarrays) [20] in combination with a nested cross validation approach [21] for their ability to independently predict the rISIS class assignment labels.

Gene set enrichment analysis (GSEA) of GO biological processes was applied to identify biological themes associated with the candidate subtypes (FDR<10%).

The validity of the rISIS subtypes was further validated in ten independent datasets (Table 1). Data from each study were normalized, probes mapped to EnsEMBL identifiers, gene expression levels robustly scaled to the range [−1, +1], and samples classified using a score based on weights determined from our linear discriminant analysis on the original dataset. Additional details of the computational methods used are provided in Text S1.

**Table 1:** Microarray datasets used as training and validation sets.

Contributors	Year	Set	Microarray platform	# Patients with ovarian tumor	# Patient with high grade, late stage, serous ovarian tumor	PMID	Source
Bentink et al.[This study]	2011	Training	Illumina DASL BeadArray 12k	129	129		E-MTAB-386
Dressman *et al.* [*5*]	2007	Validation	Affymetrix GeneChip HG-U133A	118	114	17290060	http://data.cgt.duke.edu/platinum.php
Yoshihara et al. [36]	2010	Validation	Agilent G4112A	110	43	20300634	GSE17260
Tothill *et al.* [10]	2008	Validation	Affymetrix GeneChip HG-U133A	285	139	18698038	GSE9899
Birrer *et al.* Bonome *et al.* [37]	2008	Validation	Affymetrix GeneChip HG-U133A	185	185	18593951	GSE26712
TCGATCGA [12]	2011	Validation	Affymetrix GeneChip HG-U133A	510	402	21720365	http://tcga-data.nci.nih.gov/tcga/tcgaHome2.jsp
Spentzos *et al.* [9]	2004	Validation	Affymetrix GeneChip HG-U95v2	53	41	15505275	GSE19161
Zhang *et al.* [38]	2008	Validation	Affymetrix GeneChip HG-U133PLUS2	55	46	18458333	GSE19161
Denkert *et al.* [26]	2009	Validation	Affymetrix GeneChip HG-U133A	80	41	19294737	GSE14764
Crijns *et al.* [39]	2009	Validation	Operon human v3 35K	157	85	19192944	GSE13876
Mok *et al.* [40]	2009	Validation	Affymetrix GeneChip HG-U133PLUS2	53	53	19962670	GSE18520

## Results

### Patient stratification based on 4 independent gene expression signatures

Gene expression-based stratification of cancer into transcriptionally distinct subtypes has proven to be extremely powerful in separating patients with unique clinical characteristics, and in shedding light on the genes and mechanisms responsible for driving subtype distinctions. However no robust molecular classification in ovarian has been found, despite a large number of available expression datasets. This may be due to the fact that many studies include multiple histological subtypes, reducing their power to effectively identify new molecular phenotypes [22], [23], [24], [25], [26].

To overcome the limitations of these previous studies, we generated a large gene expression data set from tumors consisting only of high grade, late stage serous carcinomas and used these for subtype discovery. We focused on high grade serous tumors as they represent, by far, the most common histologic subtype of ovarian cancer and the one most responsive to chemotherapy. We deviated from the widely-used strategy of clustering the patients based on the global similarity of their gene expression profiles as described in Tothill et al. [10] because such approaches can be cofounded by background gene expression and instead focused on finding compact modular features within the tumor expression profiles. Our approach is consistent with mechanistic models of cancer subtypes in which the expression of distinct functionally-related groups of genes and distinct pathways can define phenotypically and clinically distinct groups [27].

We used the unsupervised class discovery algorithm ISIS [15], which splits the sample set into subsets and tests the significance of the partitions using linear discriminant analysis with the 100 most significant genes. In contrast to more widely used clustering algorithms, which always return a partition of a dataset, ISIS does not report a result if the data do not support the presence of subtypes within a specified cohort.

In the analysis presented here we implemented a robust version of the ISIS algorithm, rISIS, that includes an additional bootstrap step to identify only those partitions of the samples in the test set that are not dependent on the composition of the initial training sample set. To do this rISIS chooses random subsets of the training samples, searches for subtype partitions and trains classifiers for each of these, and monitors the consistency of the predictions on the independent test samples. Only predicted partitions were consistent on the independent test samples are kept and used for further analysis.

**Figure 1:**
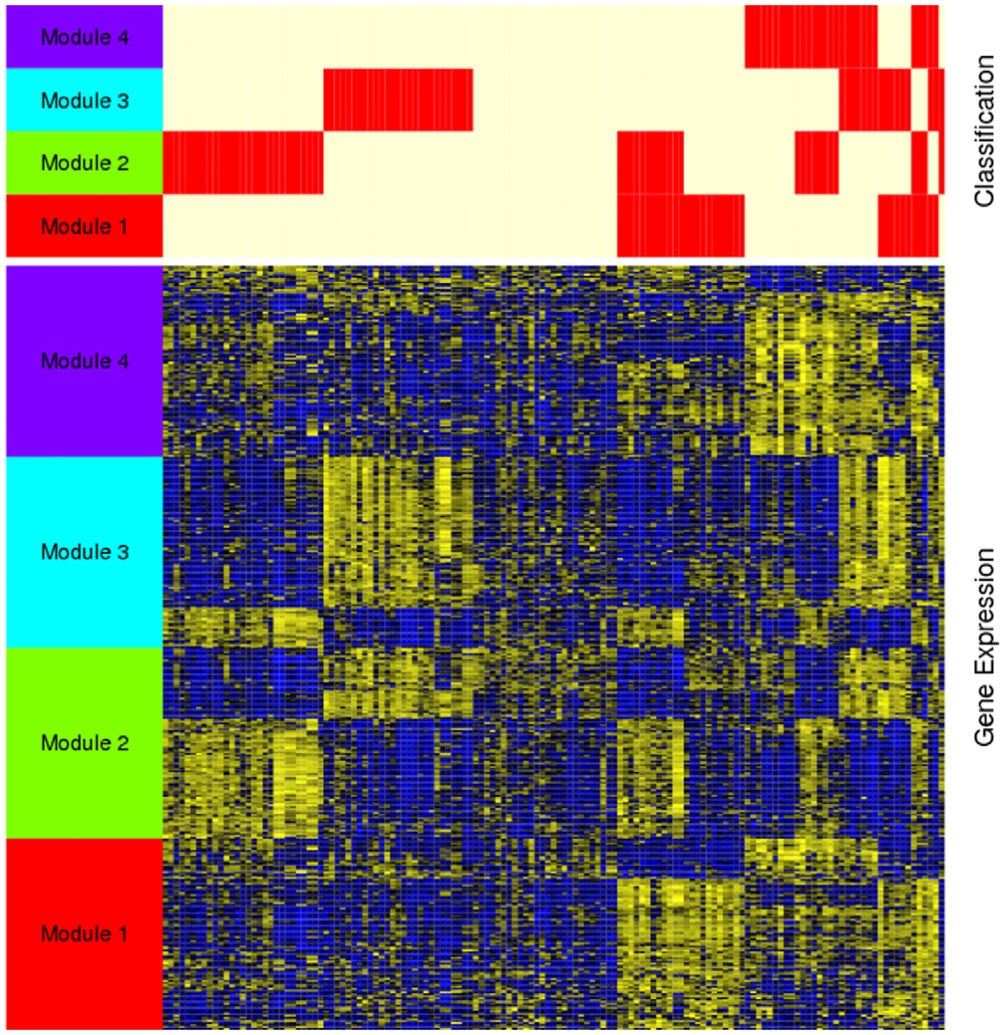
Four binary classifications of high grade ovarian serous cancer. The ISIS algorithm identified four independent binary partition classifications (splits) of 129 ovarian cancer samples. Each binary classification is supported by an independently selected set of 100 genes (module). The top panel of this figure shows four horizontal bars representing the classification of the 129 tumor samples (columns) with respect to the gene modules. Red indicates that a patient was classified into the smaller group resulting from the respective split (g1) and white indicates the classification into the larger group (g0). The heatmap in the lower panel represents the expression profiles of the gene modules supporting the four binary classifications. Each row represents a gene, each column a patient and each cell correspond to a gene and its expression level; yellow indicates an expression level of a gene above its mean across the patients and blue below its mean.

Using rISIS we found four independent, robust binary partitions, or “Splits,” in our ovarian cancer gene expression profiling data (S1–S4), each supported by expression of a defined set of 100 genes (modules) as shown in Figure 1. For each of these binary partitions, the two patient subtypes were labeled g0 and g1, for the larger and smaller subsets, respectively, so that S1 consists of S1g0 and S1g1, S2 is divided into S2g0 and S2g1 for Split2, etc. The list of genes in the modules that define each of the binary partitions is included in the File S1.

### MicroRNA expression profiles provides support for Split 1

The expression profiles for the 743 known microRNAs represented on the Illumina DASL platform [17] provided an independent source of data to test the robustness of our subtype assignments. We used Prediction Analysis of Microarrays (PAM) [20], which uses a nearest-centroid method, to test whether the nicroRNA expression was able to predict the subtype membership for each of our four binary partitions. Nested cross-validation was used to build a PAM classifier for each Split and compute an unbiased estimate of its performance [21]. As can be seen in Table 2, the microRNA expression profiles provide strongest support for the subtypes defined by Split 1; only three samples of S1g1 are misclassified within the cross-validation. The cross-validated misclassification error of S1g0 was larger, but still below 20%. Much larger misclassification error rates were observed for Splits 2–4, suggesting that these may represent either artifacts in the original mRNA expression data or subtypes of patients characterized by more complex substructure than cannot be discovered with the binary splitting approach used by rISIS.

**Table 2:** Cross-validation performance of microRNA expression based on predictors of the four binary molecular classifications (Splits 1–4).

Split 1	g0 (predicted$)	g1 (predicted$)	Error rate&	Split 2	g0 (predicted$)	g1 (predicted$)	Error rate&
g0 (true*)	80	16	**0.167**	g0 (true*)	71	12	**0.14**
g1 (true*)	3	30	**0.091**	g1 (true*)	17	29	**0.37**

Split 3	g0 (predicted$)	g1 (predicted$)	Error rate&	Split 4	g0 (predicted$)	g1 (predicted$)	Error rate&
g0 (true*)	73	17	**0.19**	g0 (true*)	89	12	**0.12**
g1 (true*)	18	221	**0.46**	g1 (true*)	8	20	**0.29**

*True labels of the 4 independent classifications identified by mRNA expression profiling.

$ Labels of the 4 independent classifications identified by miRNA expression profiling.

& Unbiased estimate of error rate of classifiers predicting Splits 1–4 from miRNA expression profiles.

The contingency tables show numbers of samples in groups defined by Splits 1–4 and predicted from miRNA expression.

Because the microRNA data provides the greatest independent support for Split 1, we chose to focus the remainder of our analysis on this subtype assignment. The consensus set microRNAs significant in classifying the samples as S1g0 or S1g1 in all cross-validation classifiers are included in File S2.

### Gene set enrichment analysis of Split 1

We then performed Gene Set Enrichment Analysis using Gene Ontology (GO) Biological Process terms to functionally characterize the gene module responsible for the Split 1 classification. GSEA significant processes included angiogenesis and extracellular matrix proteins, inducing the GO categories “vascular development” (GO:0001944; FDR = 2.5%) and “regulation of cell adhesion” (GO:0030155; FDR = 2.4%), both of which had relatively higher expression levels in S1g1, the smaller of the two classes comprising Split 1. The larger sample group, S1g0, showed relatively higher expression for genes involved in “single stranded DNA binding” (GO:0003697; FDR = 9.5%) and “structure specific DNA binding” (GO:0043566; FDR = 5.0%). Because of the significance of angiogenesis in defining the groups, we refer to S1g1 as the “angiogenic” and S1g0 as the “non-angiongenic” subtypes.

### Robustness and prognostic value of the angiogenic subtype classification

**Figure 2:**
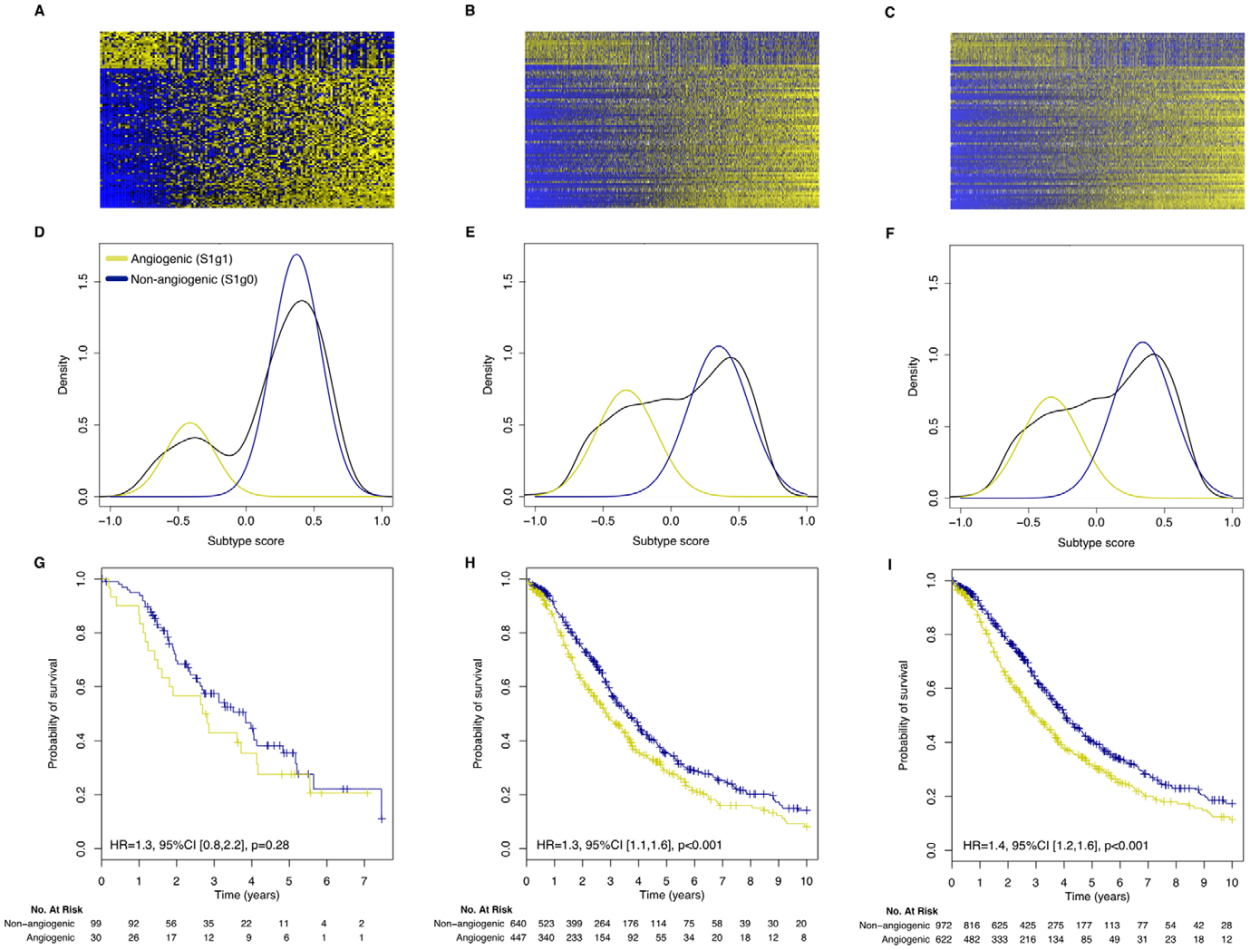
Validation of angiogenic ovarian cancer classification in our dataset and ten independent validation datasets. Panels A, D and G display the gene expression of the 100 genes used to classify ovarian tumors into angiogenic and non-angiogenic subtypes in our dataset (129 patients), the high grade, late stage, serous tumors (1,090 patients) and all tumors (1,606 patients) in the validation set, respectively. Panels D, E and F report the corresponding distribution of the scaled subtype scores. Panels B, D and F reports the (overall) survival curves of patients having tumors of angiogenic or non-angiogenic subtype in the corresponding datasets.

We tested our angiogenic subtype classification for reproducibility and association with clinical variables in our original dataset and ten previously published gene expression datasets collected on a number of diverse microarray platforms (Table 1). We normalized and scaled data from each study and assigned an, angiogenic subtype score to each of the 1,606 samples in the published gene expression datasets (see Text S1 for detailed description of the methods). The results of these assignments are shown in Figure 2 for our initial set of 129 samples (Figures 2A, 2D, 2G), for the 1,090 patients from the ten published studies having high grade (≥3), late stage (≥3), serous ovarian tumors (Figures 2B, 2E, 2H), and for all 1,606 patients in the published datasets (Figures 2C, 2F, and 2I). The top figures show heatmaps for the 100 classification genes, the middle row show the bimodal distribution of classification scores in each dataset, and the bottom row of figures shows the significantly poorer survival for the angiogenic subtype relative to the non-angiogenic subtype. An independent validation that the most robust number of subtypes in the data, estimated using the Bayesian Information Criteria (BIC), is shown in Figure S1.

Within our patient set, those with angiogenic subtype tumors had a hazard ratio (HR) = 1.3 (95%CI [0.8,2.2], logrank p-value = 0.28) relative to the non-angiogenic subtype. We saw similar, and highly significant differences for the published high-grade, late stage, serous tumors (HR = 1.3, 95%CI [1.1,1.6], logrank p-value<0.001) and the entire published ovarian dataset (HR = 1.4; 95%CI [1.2,1.6], logrank p-value<0.001). Additional plots, including classification of the ten individual published datasets can be found in Figure S2 and Figure S3.

### Association with clinical parameters

We then tested the association of the angiogenic subtype classification with the clinical information, including stage, grade, optimal debulking during surgery, and age, available for the compendium of datasets (Table 1). In our set of 129 high grade, late stage, serous ovarian cancer patients we observed no significant association with any of these clinical parameters (Fisher's exact test p-value>0.05). Similarly, in the validation set of 1,090 high-grade, late stage serous ovarian cancer patients, we found no significant association with subtype and these parameters (Fisher's exact test p-value>0.05).

However, in the validation set of 1,606 ovarian cancer patients we found significant association with grade (only 19% of grade 1 tumors are of the angiogenic subtype, Fisher's exact test p-value = 0.0025; Table 3A), stage (only 10% of stage I and II tumors are of the angiogenic subtype, Fisher's exact test p-value<0.001; Table 3B) and debulking (debulking is suboptimal for 60% versus 44% for the angiogenic and the non-angiogenic subtype tumors respectively, exact test p-value = 0.001, Table 3C). No significant association between the angiogenic subtype classification and age at diagnosis has been found.

**Table 3:** Association with clinical parameters.

A.		Grade			
		1	2	3	4
**Subtype**	Angiogenic	14	140	433	21
	Non-angiogenic	59	200	668	37

B.		Stage			
		1	2	3	4
**Subtype**	Angiogenic	4	5	520	92
	Non-angiogenic	43	39	779	115

C.		Debulking	
		Suboptimal	Optimal
**Subtype**	Angiogenic	121	81
	Non-angiogenic	143	176
	Non-angiogenic	143	176

Contingency tables for the significant association between the angiogenic subtype classification and (A) histological grade, (B) Stage, and (C) debulking in our validation set of 1,606 ovarian cancer patients. It is worth noting that different datasets are annotated using different histological grading and tumor staging systems, with scales ranging from 1 to 3 and 1 to 4. In this study, we simply merged these clinical annotations because we do not have access to the original tumor tissues to perform a standardized histological grading and tumor staging.

### Comparison with published ovarian cancer subtypes

We then compared our angiogenic subtype classification to the expression-based molecular subtypes assigned by Tothill and colleagues [24] using *k*-means clustering. These subtypes, which they called C1 (n = 83), C2 (n = 50), C3 (n = 28), C4 (n = 46), C5 (n = 36), C6 (n = 8), and NC for a set of “unassigned” cases (n = 34), were reported to correspond to different clinical and pathological features. The most significant of these was C1 which Tothill found to have significantly poor prognosis and which they described as characterized by a “reactive stroma signature.”

When we classified the Tothill samples using our angiogenic subtype classification, we observed a highly significant association with Tothill's clusters (Fisher's exact test p-value<0.001; Table S1A). Of the 115 samples classified as having the angiogenic subtype, 82 (71%) were from the C1 subtype, 18 were C2, 11 were NC, and the remaining four were from C5; none of the C3 and C4 samples were classified as angiogenic. This suggests that Tothill's C1 class is largely concordant with our angiogenic subtype.

We also compared our angiogenic subtype classification with the four subtypes (“differentiated”, “immunoreactive”, “mesenchymal”, and “proliferative”) recently defined from the gene expression dataset generated by the TCGA consortium (Table 1; [12]). These four subtypes were validated only in two datasets (TCGA and Tothill; Table 1) and did not exhibit any differences in overall survival [12]. However, we observed a significant association between our angiogenic subtype classification and TCGA's subtyping (Fisher's exact test p-value<0.001; Table S1B), where the majority of the tumors identified as angiogenic are from the TCGA mesenchymal (58%) and immunoreactive (20%) subtypes, although using our angiogenic/non-angiogenic classification they have significantly worse survival as described previously.

## Discussion

There have been many published studies that have attempted to find robust, clinically-relevant molecular subtypes in ovarian cancer, but there has been no clear consensus as to what subtypes exist. One reason for this may be that most analyses have used methods that are sensitive to subtle variations in the data, resulting in putative subtypes that cannot be generalized to independent validation datasets [28], [29]. Many studies have also used mixed histological types in their analyses, reducing the power to discover new classes and potentially confounding the results.

In the analysis presented here, we focused on a single histological type—high-grade serous ovarian cancer. We chose this because it is by far the most common histologic subtype of ovarian cancer and the one that is most responsive to chemotherapy. Moreover the vast majority of published gene expression data, including that from the TCGA, is from patients with high-grade serious cancer. Although there are other recognized ovarian histological subtypes, including mucinous, clear cell and endometrioid cancers, we did not have sufficient numbers of these to search for subtypes or to robustly validate their existence in independent datasets.

For subtype discovery, we used rISIS, a robust clustering method that combines class discovery and feature selection by searching for binary groupings of the samples that are strongly supported by statistically significant differences in gene expression [15]. Rather than relying on global patterns of expression, which can be affected by noise in the data and by the initial choice of samples, rISIS searches for separations of the initial sample set together with the genes that are significantly different between subgroups and support the separation. By design, rISIS reports multiple, overlapping partitions of the original sample set and associated classification genes, reflecting the complexity of biological systems in which there are often common pathways or functional gene groups that are activated in multiple phenotypic groups. While we found four putative subtypes partitions, each of which could be plausibly described by the genes that supported it, we were only able to confirm the existence of the first bi-partition of the samples using independently obtained microRNA expression data.

The corresponding two subtypes, defined by expression of genes associated with angiogenesis has been shown to be robust and reproducible in independently published gene expression datasets including 1,090 high grade, late stage, serious (and 1,606 total) ovarian tumors, with statistically significant differences in overall survival. The angiogenic subtype was found to be fairly concordant with Tothill's C1 “reactive stroma” cluster [24] and to overlap with mesenchymal and immunoreactive subtypes identified by the TCGA consortium [12], but with better statistical support and a well-defined, functionally-associated set of classification gene modules. When we extended our analysis to all 1,606 patients, which include low grade and early stage patients, the bimodality of the subtype score was preserved and the association with survival was even stronger. Overall, this suggests that the angiogenic subtype represents a true biological subset of serous ovarian tumors that can be robustly identified across independent datasets.

This identification of an angiogenesis-driven subtype is of potential clinical and translational importance since several anti-angiogenic agents (bevacizumab, cediranib) are being added to chemotherapy both in newly diagnosed patients and those with recurrent cancer. and inhibitors of DNA repair (Olaparib) [30], [31], [32].

Bevacizumab is a humanized monoclonal antibody that recognizes circulating vascular endothelial growth factor (VEGF) and which has documented anti-cancer activity patients with recurrent ovarian cancer [30], [31], [32], [33], [34], [35]. Single agent bevacizumab demonstrates response rates of 18–20% suggesting activity of this anti-angiogenic in ovarian cancer [30], [31]. In these studies, bevacizumab has been identified as an active drug in both platinum-resistant and platinum-sensitive recurrent ovarian cancer.

In Gynecologic Oncology Group study 218, bevacizumab was added to upfront chemotherapy for newly diagnosed patients with advanced ovarian cancer, and the study design was comprised of 3 groups: carboplatin and paclitaxel given IV with placebo during chemotherapy and maintenance, carboplatin/paclitaxel/bevacizumab with placebo maintenance, and carboplatin/paclitaxel/bevacizumab followed by 12 months of bevacizumab maintenance. A statistically significant improvement of 4 months was observed in the carboplatin/paclitaxel/bevacizumab plus bevacizumab maintence arm compared to carboplatin/paclitaxel alone [33].

ICON7 also demonstrated a statistically significant improvement in PFS with the addition of bevacizumab to upfront chemotherapy [35]. In patients with platinum sensitive recurrent ovarian cancer, the addition of bevacizumab to carboplatin and gemcitabine chemotherapy statistically improved PFS compared to carboplatin and gemcitabine chemotherapy alone [34]. Thus, increasing clinical data in both newly diagnosed and recurrent ovarian cancer suggests some benefit in PFS with the addition of bevacizumab [34], [35].

Given the important toxicities of these agents such as gastrointestinal bowel perforations, thromboembolic events such as strokes and pulmonary emboli, and hypertension, as well as their financial cost, a classification signature that could identify and pre-select patients whose tumors would most likely benefit from receipt of anti-angiogenic agents would be of tremendous clinical importance. A retrospective assessment of gene expression profiles of patients in the treatment arm of one of these clinical trials would help establish the validity of this signature for predicting relevant response.

There may be additional translational applications of our angiogenic/non-angiogenic subtype assignments. The classification gene set also was enriched for genes associated with the extracellular matrix and a number of agents that interfere with the ECM, including notch inhibitors and integrin modulators, are currently being tested in ovarian cancer. Again, a retrospective analysis of gene expression data from patients in the treatment arms of these trials could help to establish a clinical application for the subtypes we identified.

Although much remains to be done, we have developed a new approach to robust subtype discovery, demonstrated its application in disease in which subtype identification has proven challenging, and validated that our subtype assignments are robust in a large independent dataset—and more strongly predictive of outcome than any previously reported signature in ovarian cancer. Given the overlap between the genes that drive the subtype classification, and the clinical trials underway in ovarian cancer, we believe our classification has great potential to help influence treatment and improve outcomes for patients.

## Supporting Information

Figure S1 **Identification of the most likely number of Gaussians to model the subtype scores in the training set (** **Figure 1A–B** **) and in the validation set for the 1,090 patients having high grade (≥3), late stage (≥3), serous ovarian tumors (** **Figure 1C–B** **) and for all the 1,606 patients (** **Figure 1E–F** **).** Panel A displays the distribution of the subtype scores and the mixture of two Gaussians, what is the most likely model given the data as estimated by the Bayesian Information Criterion (BIC) in panel B. As can be seen in panels C and E, the distribution of the subtype scores still exhibits a bimodal pattern despite the heterogeneity of the validation datasets (different microarray technologies and normalization methods); this is confirmed by the estimation of the BIC in the validation (panels D and F) where a mixture of two or three Gaussians are the most likely models given the data.
(DOCX)

Figure S2 **Subtype identification for high grade, late stage, serous ovarian tumors in each of the ten independent validation sets separately: from left to right, a short description of the dataset is given along with the number of tumors in the angiogenic and non-angiogenic subtypes, the bimodal distribution of the rescaled subtype scores, and the survival curves of patients having an angiogenic of non-angiogenic subtype.**
(DOCX)

Figure S3 **Subtype identification for all tumors (all grades, all stages, all histological types) in the training set and in each of the ten independent validation sets separately: from left to right, a short description of the dataset is given along with the number of tumors in the angiogenic and non-angiogenic subtypes, the bimodal distribution of the rescaled subtype scores, and the survival curves of patients having an angiogenic of non-angiogenic subtype.**
(DOCX)

Table S1 **Association between the angiogenic subtype classifications and (A) the putative subtype clusters in Tothill's dataset, and (B) the proposed subtypes in the ovarian TCGA dataset.**
(DOCX)

Text S1 **Angiogenic mRNA and microRNA gene expression signatures predict a novel subgroup of serous ovarian cancer.**
(DOCX)

File S1 **An Excel workbook containing the list of genes in the modules that define each of the four binary partitions found using rISIS, with each represented in separate tab.**
(XLSX)

File S2 **An Excel spreadsheet containing the consensus set microRNAs significant for classifying the samples as S1g0 or S1g1 in all cross-validation classifiers.**
(XLSX)
