# Supplementary material for: Angiogenic mRNA and microRNA Gene Expression Signature Predicts a Novel Subtype of Serous Ovarian Cancer
Source: PLoS One. 2012 Feb 13;7(2):e30269. doi: 10.1371/journal.pone.0030269 (PMC3278409; doi:10.1371/journal.pone.0030269)
Supplement: Figure S1 — Identification of the most likely number of Gaussians to model the subtype scores in the training set ( Figure 1A–B ) and in the validation set for the 1,090 patients having high grade (≥3), late stage (≥3), serous ovarian tumors ( Figure 1C–B ) and for all the 1,606 patients ( Figure 1E–F ). Panel A displays the distribution of the subtype scores and the mixture of two Gaussians, what is the most likely model given the data as estimated by the Bayesian Information Criterion (BIC) in panel B. As can be seen in panels C and E, the distribution of the subtype scores still exhibits a bimodal pattern despite the heterogeneity of the validation datasets (different microarray technologies and normalization methods); this is confirmed by the estimation of the BIC in the validation (panels D and F) where a mixture of two or three Gaussians are the most likely models given the data. (DOCX) [file pone.0030269.s001.docx]

**Supplemental Figure S1** – Identification of the most likely number of Gaussians to model the subtype scores in the training set (Figure 1A-B) and in the validation set for the 1,090 patients having high grade (≥3), late stage (≥3), serous ovarian tumors (Figure 1C-B) and for all the 1,606 patients (Figure 1E-F). Panel A displays the distribution of the subtype scores and the mixture of two Gaussians, what is the most likely model given the data as estimated by the Bayesian Information Criterion (BIC) in panel B. As can be seen in panels C and E, the distribution of the subtype scores still exhibits a bimodal pattern despite the heterogeneity of the validation datasets (different microarray technologies and normalization methods); this is confirmed by the estimation of the BIC in the validation (panels D and F) where a mixture of two or three Gaussians are the most likely models given the data.
