# Supplementary material for: Angiogenic mRNA and microRNA Gene Expression Signature Predicts a Novel Subtype of Serous Ovarian Cancer
Source: PLoS One. 2012 Feb 13;7(2):e30269. doi: 10.1371/journal.pone.0030269 (PMC3278409; doi:10.1371/journal.pone.0030269)
Supplement: Figure S3 — Subtype identification for all tumors (all grades, all stages, all histological types) in the training set and in each of the ten independent validation sets separately: from left to right, a short description of the dataset is given along with the number of tumors in the angiogenic and non-angiogenic subtypes, the bimodal distribution of the rescaled subtype scores, and the survival curves of patients having an angiogenic of non-angiogenic subtype. (DOCX) [file pone.0030269.s003.docx]

**Supplemental Figure S3**– Subtype identification for all tumors (all grades, all stages, all histological types) in the training set and in each of the ten independent validation sets separately: from left to right, a short description of the dataset is given along with the number of tumors in the angiogenic and non-angiogenic subtypes, the bimodal distribution of the rescaled subtype scores, and the survival curves of patients having an angiogenic of non-angiogenic subtype.
