# Supplementary material for: Angiogenic mRNA and microRNA Gene Expression Signature Predicts a Novel Subtype of Serous Ovarian Cancer
Source: PLoS One. 2012 Feb 13;7(2):e30269. doi: 10.1371/journal.pone.0030269 (PMC3278409; doi:10.1371/journal.pone.0030269)
Supplement: Table S1 — Association between the angiogenic subtype classifications and (A) the putative subtype clusters in Tothill's dataset, and (B) the proposed subtypes in the ovarian TCGA dataset. (DOCX) [file pone.0030269.s004.docx]

# Supplemental Tables

**Supplemental Table S1 –** Association between the angiogenic subtype classifications and (A) the putative subtype clusters in Tothill’s dataset, and (B) the proposed subtypes in the ovarian TCGA dataset.

**A**

|  |  | **Tothill's subtyping** | | | |  |  |  |
| --- | --- | --- | --- | --- | --- | --- | --- | --- |
|  |  | C1 | C2 | C3 | C4 | C5 | C6 | NC |
| **Angiogenic subtyping** | Angiogenic | 82 | 18 | 0 | 0 | 4 | 0 | 11 |
|  | Non-angiogenic | 1 | 32 | 28 | 46 | 32 | 8 | 23 |

**B**

|  |  | **TCGA's subtyping** | | | |
| --- | --- | --- | --- | --- | --- |
|  |  | Differentiated | Immunoreactive | Mesenchymal | Proliferative |
| **Angiogenic subtyping** | Angiogenic | 26 | 36 | 104 | 14 |
|  | Non-angiogenic | 109 | 70 | 3 | 123 |
