# Supplementary material for: Angiogenic mRNA and microRNA Gene Expression Signature Predicts a Novel Subtype of Serous Ovarian Cancer
Source: PLoS One. 2012 Feb 13;7(2):e30269. doi: 10.1371/journal.pone.0030269 (PMC3278409; doi:10.1371/journal.pone.0030269)
Supplement: Text S1 — Angiogenic mRNA and microRNA gene expression signatures predict a novel subgroup of serous ovarian cancer. (DOCX) [file pone.0030269.s005.docx]

# Supplemental Information

# Angiogenic mRNA and microRNA gene expression signatures predict a novel subgroup of serous ovarian cancer

Stefan Bentink^1,6,*^, Benjamin Haibe-Kains^1,6,*^, Thomas Risch^1^, Jian-Bing Fan^3^, Michelle S. Hirsch^4,7^, Kristina Holton^1^, Renee Rubio^1^, Craig April^3^, Jing Chen^3^, Eliza Wickham-Garcia^3^, Joyce Liu^2,7^, Aedin Culhane^1,6^, Ronny Drapkin^4,5,7^, John Quackenbush^1,2,6,$^, and Ursula A. Matulonis^5,7,$^

**^*^ Co-first authors**

**^$^ Co-last authors**

^1^Department of Biostatistics and Computational Biology, Dana-Farber Cancer Institute, Boston, MA 02115, ^2^Department of Cancer Biology, Dana-Farber Cancer Institute, Boston, MA 02115, ^3^Illumina, Inc., San Diego, CA 92121, ^4^Department of Pathology, Division of Woman's and Perinatal Pathology, Brigham and Women’s Hospital, Boston, MA 02115, ^5^Department of Medical Oncology, Dana-Farber Cancer Institute, Boston, MA 02115, ^6^Harvard School of Public Health, Boston MA 02215, ^7^Harvard Medical School, Boston, MA 02215

# Supplemental Methods

## Subtype discovery

We split our cohort of 129 tumor samples into a training set (n=82) and a model selection set (n=47). Twelve samples from the training set were arrayed in duplicate, so that we had 12,449 normalized gene expression values in 141 microarray assays. The profiled genes were filtered to identify the 1,000 that were most variable across samples, yet most reproducible among replicates. To do this, we first computed on a gene per gene basis the ratio of the variance across the training samples and the variance within the replicated samples; large values of this ratio indicate high biological and low technical variability of gene expression measurements.

To identify binary partitions (splits) of the ovarian cancer samples into potential tumor subgroups, we ran ISIS class discovery [[1](#_ENREF_1)] on gene expression profiles of 1000 filtered genes in 82 training samples. We chose the default parameters for ISIS, except we set p=100 (the number of genes supporting each bi-partions), and p_offs_=5 (number of top differentially expressed genes not included in the computation of the diagonal linear discriminant score). For samples with replicate gene expression profiles, one of the two assays was selected at random for class discovery.

From the resulting large set of potential binary classifications we selected those that were most robust with respect to random bootstrap perturbations of the training set. For our bootstrap analysis, we performed 1000-folds in which,we randomly chose, with replacement, 82 patients from the set of training samples, selected the 100 most significant differentially expressed genes ranked by the absolute value of a two sample t-statistic computed for each gene between the sample classes (angiogenic vs non-angiogenic) and learned a diagonal linear discriminant classifier .We applied the 1000 bootstrap classifiers to the 47 samples in the model selection set, yielding 1000 predictions for each bi-partition on each of the 47 samples.

We defined the consistency of class predication as the relative number of times the samples in the model selection set were mapped to the same class by the 1000 bootstrapped classifiers. From the large set of ISIS bi-partitions, we used only those with at least 95% consistency across all bootstrap perturbations; this yielded four robust bi-partitions of the samples that we used for the remainder of our analysis (see Results). The resulting list of 100 genes and their corresponding weight, which are used to identify the subtypes, are given in Supplemental File 1.

## Validation on independent data sets

To validate the ISIS classification results on independent data sets we collected ten publicly available microarray datasets from Gene Expression Omnibus (GEO; [[2](#_ENREF_2)]) and authors’ websites (see Table 1 in the main manuscript). This compendium of datasets comprises 1,606 ovarian cancer patients, 1,090 of whom were classified as having a high grade, late stage, serous ovarian tumor. When Affymetrix GeneChip HG-U133A and PLUS2 have been used, the CEL files were normalized with fRMA [[3](#_ENREF_3)]. For Affymetrix HG-U92v2 (dataset published in Spentzos et al, 2004) we used RMA [[4](#_ENREF_4)] to normalize raw expression measurements while we retrieved the normalized gene expression data published on authors’ website for Yoshihara et al, and Crijns et al.

For each microarray platform, probes were mapped to EnsEMBL identifiers using the *biomaRt* Bioconductor package version 2.8.1[[5](#_ENREF_5)]. When multiple probes on an array mapped to the same EnsEMBL identifier, we used the probe with the highest variance as the representative of that gene.

To identify ovarian cancer molecular subtypes in these diverse datasets we used a simple yet robust subtype score defined by:

$$s_{j}=\frac{\sum_{i\in I} w_{i}g_{ij}}{\sum_{i\in I} |w_{i}|}$$

where *s_j_* is the subtype score for tumor *j*, *I* is the set of classification genes (Supplemental File 1) that can be mapped to the microarray platform used for the samples being classified, *g_ij_* is the normalized expression for gene *i* in tumor *j* and *w_i_* is the weight of gene *i* in *I* calculated using linear discriminant analysis in our original training set.

To allow comparison between datasets, we applied a robust linear scaling to the subtype scores such that quantiles 2.5% and 97.5% of the subtype scores for the high grade, late stage, serous tumors in each dataset were set to -1 and +1 respectively using the *genefu* Bioconductor package version 1.2.1 [[6](#_ENREF_6)]. This scaling has been shown to improve the consistency of prediction scores between classifiers in breast cancer datasets using diverse microarray technologies and normalization procedures [[7](#_ENREF_7),[8](#_ENREF_8)]. The rescaled subtype scores for all tumors in our compendium of datasets are provided in Supplemental File 3.

As can be seen in Figure 2A in the main manuscript and in Supplementary Figure 1A, the resulting subtype scores had a bimodal distribution and so we fitted a mixture a two Gaussians using the Mclust R package version 3.4.10, enabling us to compute the posterior probability that each tumor belongs to the angiogenic and non-angiogenic subtypes given its subtype score [[9](#_ENREF_9)]. We then classified each tumor as belonging to one of the two subtypes based on their maximum posterior probability (see Figure 2 in the main manuscript and Supplemental Figures 1-3 for the distribution of the subtype scores in the training set and the validation set).

# Supplemental Files

**Supplemental File 1** ‘supplemental_file1.xlsx’ – Spreadsheet containing the list of 100 genes and their corresponding weights as estimated by ISIS for Split 1, 2, 3, and 4.

**Supplemental File 2** ‘supplemental_file2.xlsx’ – Spreadsheet containing the rescaled subtype scores and clinical information for all tumors in our compendium of training and validation datasets.

**Supplemental File 3** ‘supplemental_file3.xlsx’ – Spreadsheet containing the rescaled subtype scores and clinical information for all tumors in our compendium of training and validation datasets.

# Reproducibility

A complete documentation of the analysis and the results including the complete R-code ([www.r-project.org](http://www.r-project.org)) can be found on a supplemental website (<http://compbio.dfci.harvard.edu/pubs/ovarian_expression/>)

# References

1. von Heydebreck A, Huber W, Poustka A, Vingron M (2001) Identifying splits with clear separation: a new class discovery method for gene expression data. Bioinformatics 17 Suppl 1: S107-114.

2. Gorlov IP, Meyer P, Liloglou T, Myles J, Boettger MB, et al. (2007) Seizure 6-like (SEZ6L) gene and risk for lung cancer. Cancer Res 67: 8406-8411.

3. McCall MN, Bolstad BM, Irizarry RA (2010) Frozen robust multiarray analysis (fRMA). Biostatistics (Oxford, England) 11: 242-253.

4. Irizarry RA, Hobbs B, Collin F, Beazer-Barclay YD, Antonellis KJ, et al. (2003) Exploration, normalization, and summaries of high density oligonucleotide array probe level data. Biostatistics (Oxford, England) 4: 249-264.

5. Smedley D, Haider S, Ballester B, Holland R, London D, et al. (2009) BioMart – biological queries made easy. BMC genomics 10: 22.

6. Haibe-Kains B, Schroeder M, Culhane AC, Bontempi G, Sotiriou C, et al. (2011) GeneFu: Relevant Functions for Gene Expression Analysis, Especially in Breast Cancer. R/Bioconductor.

7. Haibe-Kains B, Culhane AC, Desmedt C, Bontempi G, Quackenbush J, et al. (2011) A three-gene model to robustly identify breast cancer molecular subtypes. submitted.

8. Haibe-Kains B, Desmedt C, Rothé F, Piccart M, Sotiriou C, et al. (2010) A fuzzy gene expression-based computational approach improves breast cancer prognostication. Genome biology 11: R18.

9. Fraley C, Raftery AE (2002) Model-Based Clustering, Discriminant Analysis, and Density Estimation. Journal of the American Statistical Association 97: 611-631.
